# Parents’ prioritised outcomes for trials investigating treatments for paediatric severe infection: a qualitative synthesis

**DOI:** 10.1136/archdischild-2019-316807

**Published:** 2019-06-07

**Authors:** Kerry Woolfall, Caitlin O’Hara, Elizabeth Deja, Ruth Canter, Imran Khan, Paul Mouncey, Anjali Carter, Nicola Jones, Jason Watkins, Mark David Lyttle, Lyvonne Tume, Rachel Agbeko, Shane M Tibby, John Pappachan, Kent Thorburn, Kathryn M Rowan, Mark John Peters, David Inwald

**Affiliations:** 1 Health Services Research, University of Liverpool, Liverpool, UK; 2 Intensive Care National Audit & Research Centre (ICNARC), London, UK; 3 Centre of Primary Care and Public Health, Queen Mary University of London, London, UK; 4 Patient partner, London, UK; 5 Emergency Department, University Hospitals Bristol NHS Foundation Trust, Bristol, UK; 6 Health and Applied Sciences, University of the West of England, Bristol, UK; 7 Paediatric Intensive Care, Great North Children’s Hospital, Newcastle upon Tyne, UK; 8 Department of Paediatric Intensive Care, Evelina Childrens Hospital, Guys St Thomas NHS Foundation Trust, London, UK; 9 Paediatric Intensive Care, Southampton Children’s Hospital, Southampton, UK; 10 Paediatric Intensive Care, Royal Liverpool Childrens Hospital Alder Hey, Liverpool, UK; 11 Paediatric Intensive Care, Great Ormond St Hospital NHS Trust, London, UK; 12 Paediatric Intensive Care Unit, Imperial College Healthcare NHS Trust, London, UK

**Keywords:** paediatrics, outcomes research, severe infection, clinical trials, parents

## Abstract

**Objective:**

To identify parents’ prioritised outcomes by combining qualitative findings from two trial feasibility studies of interventions for paediatric suspected severe infection.

**Design:**

Qualitative synthesis combining parent interview data from the Fluids in Shock (FiSh) and Fever feasibility studies. Parents had experience of their child being admitted to a UK emergency department or intensive care unit with a suspected infection.

**Participants:**

n=: 85 parents. FiSh study: n=41 parents, 37 mothers, 4 fathers, 7 were bereaved. Fever study: n=44 parents, 33 mothers, 11 fathers, 7 were bereaved.

**Results:**

In addition to survival, parents prioritised short-term outcomes including: organ and physiological functioning (eg, heart rate, breathing rate and temperature); their child looking and/or behaving more like their normal self; and length of time on treatments or mechanical support. Longer term prioritised outcomes included effects of illness on child health and development. We found that parents’ prioritisation of outcomes was influenced by their experience of their child’s illness, survival and the point at which they are asked about outcomes of importance in the course of their child’s illness.

**Conclusions:**

Findings provide insight into parent prioritised outcomes to inform the design of future trials investigating treatments for paediatric suspected or proven severe infection as well as core outcome set development work.

**What is already known on this topic?**

- The evaluation of interventions for the treatment of children with suspected or proven severe infection has been hampered by the underdevelopment of outcome measures.
- The selection of clinical trial outcome measures is predominantly led by clinicians and researchers rather than by patients and their families.
- Research is needed to ensure the selection of outcomes in trials investigating treatments for paediatric severe infection reflects the priorities of families.

**What this study adds?**

- Our synthesis showed that in addition to survival, parents prioritised short-term outcomes including: organ and physiological functioning (eg, heart rate, breathing rate and temperature); their child looking and/or behaving more like their normal self; and length of time on treatments or mechanical support.
- Longer term prioritised outcomes included effects of illness on child health and development.
- Parents’ prioritisation of outcomes was influenced by their experience of their child’s illness, survival and the point at which they are asked about outcomes of importance in the course of their child’s illness.

## Introduction

Severe infections remain a major cause of mortality and morbidity in paediatric clinical care.^1^ International research prioritisation exercises^2–4^ highlight the need for research to explore which interventions may improve patient outcomes for paediatric severe infection. However, little knowledge exists about which outcomes are most important to parents of children treated for severe infection requiring fluid resuscitation and/or intensive care admission.^5 6^

Despite recognition that patients and the public have a legitimate and requisite role in the design and conduct of health research, the extent to which this occurs is less clear.^7 8^ Two systematic reviews^7 9^ of outcome measures in clinical trials suggest that they are predominantly selected by clinicians and researchers. Sinha *et al* 9 observed that only 3 out of 13 groups who had published on the selection of outcomes for paediatric clinical trials had consulted parents; none involved children.

To inform the design of two trials involving children with suspected severe infection, we used qualitative research to explore parents’ views on trial acceptability and important treatment outcomes. This paper presents combined qualitative findings from the Fluids in Shock (FiSh) and Fever studies (see table 1) on parents’ prioritised outcomes.

**Table 1:** Overview of the Fluids in Shock (FiSh) and Fever feasibility study designs

FiSh		Fever study
Aims	To establish the feasibility of conducting a randomised controlled trial comparing restrictive fluid bolus therapy (10 mL/kg) with the current UK recommended practice (20 mL/kg).	To establish the feasibility of conducting a definitive trial comparing temperature thresholds at which staff deliver antipyretic intervention in critically ill children with fever due to infection (intervention treat at ≥39.5°C, standard care treat at >37.5°C).
Feasibility study elements	(1) Qualitative study exploring parent views. (2) Nine-month pilot trial with integrated parent and staff perspectives study.	(1) Qualitative study exploring parent and clinician views. (2) Observational study of the epidemiology of fever in children with infection in paediatric intensive care unit. (3) Four-month pilot trial with integrated parent and staff perspectives study.
Pilot trial participants	Children (age >37 weeks (corrected gestational age) and <16 years) admitted to an emergency department with clinical suspicion of infection and signs of shock after receipt of 20 mL/kg of bolus fluid.	Children (age >29 days and <16 years) admitted to intensive care with a fever (≥37.5°C) in the first 48 hours and suspected infection who require mechanical ventilation.

## Methods

FiSh and Fever studies took place between 2015 and 2018. Both began with a qualitative feasibility study, led by the same qualitative research team (phase 1). This involved semistructured interviews with parents (including bereaved parents) of children who had attended UK emergency departments (EDs) or were admitted to a paediatric intensive care unit (PICU) with severe infection in the previous 3 years. Phase 1 informed the design of pilot trials (phase 2; 9-month recruitment period in FiSh and 4 months in Fever), which had an integrated interview element involving parents of randomised patients.

We used previous research^10–12^ to develop interview topic guides to explore parents’ views on trial design and conduct,^13^ including outcomes for the proposed definitive trials (see online supplementary table S1). To inform discussions with parents (phase 1), we conducted a literature review to develop a list of outcome measures previously reported in paediatric severe infection research.

10.1136/archdischild-2019-316807.supp1 Supplementary data

### Recruitment and interview conduct

**Figure 1:**
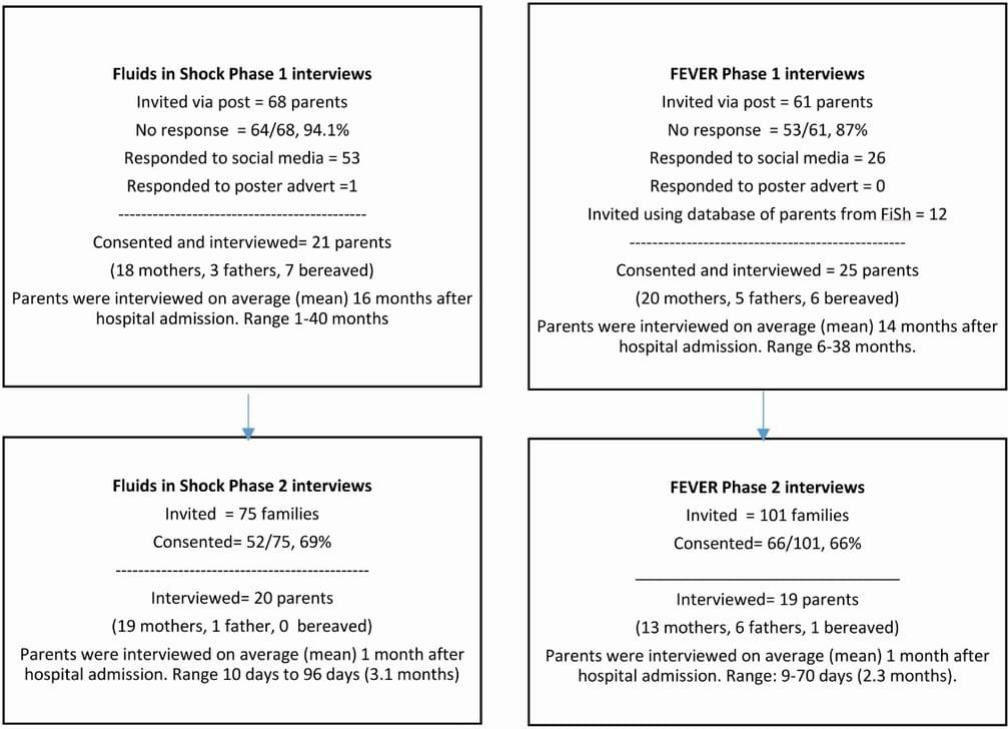
Participant recruitment and sample characteristics by study and phase.

Based on previous studies^14 15^ we anticipated needing 15–25 parents for each qualitative study phase (see figure 1). In phase 1 studies, parents were recruited through postal contact, advertising in EDs and PICUs and online including social media.^13^ A database of FiSh potential participants who consented to contact about future studies but had not taken part in an interview in FiSh were approached to participate in Fever. In phase 2 studies, clinicians provided parents with information and sought consent to contact for interviews as part of the FiSh and Fever pilot trial consent discussion.

In phase 1, expressions of interest to participate were responded to sequentially. Selection of parents for interview aimed to ensure variance of recruitment method (eg, social media, postal/database invitation and poster advert). The list of potential outcomes was emailed to parents to read prior to interview. Verbal consent was audio recorded. In phase 2, CO, ED or KW contacted parents within 1 month of consent or hospital discharge. Selection of parents for interview aimed to include all pilot sites and range of parent experience (eg, consented and declined the trial and trial arm allocation). Consent for audio recording was confirmed. In both phases, parents were asked to reflect on their personal experiences to identify important outcomes for future trials (see online supplementary table S1). Screening and interviews stopped on reaching data saturation (see online S2 box 2 for a glossary of terms).^16 17^

10.1136/archdischild-2019-316807.supp2 Supplementary data

A professional transcription company (Voicescript Ltd, Bristol, UK) transcribed verbatim digital audio recordings. Transcripts were anonymised and checked for accuracy.

### Data analysis and synthesis

Psychologists CO (PhD, female research associate FiSh) and ED (PhD, female research associate Fever) led the analysis with assistance and oversight from KW (sociologist). Analysis for each study was broadly interpretive, inductive and iterative^14 18^ as informed by the constant comparative approach.^19–22^ Outcomes were identified through direct questioning as well as referred to indirectly by parents during wider interview discussion.

NVivo V.10 software (QSR International Pty Ltd, Melbourne, Australia) was used to assist in the organisation and coding of data. CO, ED and KW met regularly to discuss interpretation and develop the coding framework. To present a prioritised list of outcomes for each study phase, we listed outcomes in order of how commonly they were discussed by parents. Outcomes discussed through direct questioning were placed higher in the list than outcomes identified in general interview discussions. Lists for FiSh and Fever phases 1 and 2 were then merged.

## Results

### Sample

We interviewed 85 parents (82 via telephone, 3 face to face), including 41 (37 mothers, 4 fathers) from the FiSh study and 44 (33 mothers, 11 fathers) from the Fever study (figure 1). Only the researchers and participants were present. Fourteen parents were bereaved (13 from phase 1 studies and 1 from phase 2). We reached data saturation point^16 17^ at 21 (FiSh) and 25 (Fever) interviews in phase 1 and 20 (FiSh) and 19 (Fever) interviews in phase 2. Due to the different recruitment designs, parents in phase 1 studies were interviewed at a later time point after their child’s hospital admission than parents in phase 2 studies (see figure 1). Interviews took between 30 min and 55 min.

### Outcomes of importance to parents of children with severe infection

Many outcomes discussed by parents during interviews mapped closely to outcomes identified in the literature review. Parents often prioritised multiple outcomes (eg, in FiSh phase 1, an average of six outcomes, and seven outcomes in phase 2).

Online table 2 (S3) shows outcomes identified in the analysis of FiSh and Fever interview transcripts. Outcomes are ranked in order of importance, defined as how many parents mentioned a particular outcome when asked directly which indicators were most important to them.

10.1136/archdischild-2019-316807.supp3 Supplementary data

Parents did not always view treatment outcomes for severe infection as a set of independent constructs. Many described important indicators of improvement as lying on a continuum, or ‘*gradient of seriousness*’ (P6, mother, non-bereaved, FiSh phase 1). Initially looking for ‘*the worst [outcome] and then… you sort of progressively aim towards the sort of next hurdle to get over*’ (P10, mother, non-bereaved, FiSh phase 1).

The most commonly prioritised outcomes are presented with illustrative quotations in table 2.

**Table 2:** Illustrative quotes for the top five prioritised outcomes

Prioritised outcome	Illustrative quotes from FiSh and Fever
*Improvement in organ and physiological function*	‘*The organ dysfunction was a big thing*’ (P17, mother, bereaved, FiSh phase 1). ‘*Probably things like heart rate coming down, blood pressure going up, his temperature improving, which it did very quickly’* (P41, mother, non-bereaved, FiSh phase 2). ‘*It would be things like his vital signs being normal, um, so obviously temperature, breathing, heart rate*’ (P01, mother, non-bereaved, Fever phase 1). ‘*So it’s taking the measures really that the nurses are looking at, like temperature, erm, infection markers, erm, heart rate, things like that. You get glued to the screens which have all the information on*’ (P82, mother, non-bereaved, Fever phase 2).
*Looking and behaving more like normal self*	‘*When they did wake him up he was alert and he could talk to his sister. So you know, as far as I was concerned he was actually getting over it, he’d sorted the chest infections, it was the secondary one that got him unfortunately*’ (P21, mother, bereaved, FiSh phase 1). ‘*I wanted his […] rash to stop spreading all over his body… It was spreading rapidly all over his body and his face was swelling. It was the most scariest thing to ever witness*’ (P40, mother, non-bereaved, FiSh phase 2). *‘The fact that they’re more with it, um more alert. Um, ‘cause like with (child name) you know when he’s getting better ‘cause he’s got a cheeky little smile. Every parent will know in themselves when their child is getting better, um, wanting to get out, wanting to sit up, you know, having something to eat and drink’* (P11, mother, non-bereaved, Fever phase 1). ‘*Erm, that he’s, erm, behaving more himself, so he’s maybe not, not the same as… not so lethargic, erm, not warm to the touch, not clammy, sweaty, and sort of returning to his normal behaviours*’(P80, mother, non-bereaved, Fever phase 2).
*Long-term effects (eg, health and development)*	‘*I think developmental sort of outcomes after is quite important, you know, whether it’s had a long term effect on them*’ (P8, mother, non-bereaved, FiSh phase 1). ‘*The thing I was most worried about when, um, when we left hospital was any long-term effects. Because obviously I mean she was less than five weeks old, she’d, she hadn’t stopped breathing entirely but she’d, um, she wasn’t taking as much, many breaths as she should have done, so I was really worried about would there be any long-term development problems with her’* (P25, mother, non-bereaved, Fever phase 1).
*Length of hospital stay*	‘*Definitely length of ICU stay is an important one, length of hospital stay certainly*’ (P01, mother, non-bereaved, FiSh phase 1). ‘*Reduce the stay in ICU, would probably be the main one*’ (P08, mother, non-bereaved, Fever phase 1).
*Reduce need/time spent on treatments or mechanical support*	‘*The length of time that he was sedated, which was as a result of the length of time that he needed mechanical breathing and things like that*’ (P7, father, non-bereaved, FiSh phase 1). ‘*The fewer machines that were operating with her. I mean we’d go from having 12 machines running or 12 syringes going in one shape or form. When the syringes got removed and they’d have six or four, you knew things were improving*’ (P39, mother, non-bereaved, FiSh phase 2). ‘*Less reliance on, um, life support equipment so, you know, more breathing on his own and not having to be on dialysis or, um, er, um, and him not having, not having to be, er, as heavily sedated as well*’ (P08, mother, non-bereaved, Fever phase 1). ‘*How quickly they’re able to come off intubation*’ (P53, father, non-bereaved, Fever phase 2).
Survival	‘*Well, yeah, obviously survival should be at the top ‘cause that’s the, that’s the big one, isn’t it?*’ (P08, mother, non-bereaved, Fever phase 1). ‘Survival *is the main, it is the main thing*’ (P78, mother, non-bereaved Fever phase 2).

FiSh, Fluids in Shock.

#### Improvement in organ and physiological function

Thirty-eight parents (38/85, 45%) described the importance of noticeable improvements in their child’s organ functioning and core physiological parameters as short-term treatment outcomes. These included heart rate, temperature, dehydration, blood pressure and respiratory rate.

#### Looking and behaving like normal self

The concept of a general, overall feeling of return to health or normality was referred to by 33/85 (40%) parents. Parents described this as an improvement in their child’s appearance and temperament. For those with sepsis, this included a reduction in the magnitude of swelling and skin discolouration. Improvements in child temperament included references to behaviour and mood, including increased alertness and decreased irritability. These outcomes were linked to improvement in other core functions of life, including ‘*feeding*’ (P49, mother, non-bereaved, Fever phase 2), ‘*talking’* (P19, mother, non-bereaved, FiSh phase 2) and ‘*walking’* (P20, mother, non-bereaved, FiSh phase 1). Together, improvement in these symptoms was taken as an important indicator to parents that their child was recovering and on the road to ‘*looking more like herself*’ (P13, mother, non-bereaved, FiSh phase 1).

#### Long-term health and development

Parents of recovered children (22/71, 31%) described how their child’s health status following hospital discharge was an important indicator of treatment efficacy. Prioritised outcomes included global development and functioning and quality of life. Health status was almost always spoken of in terms of the overall impact it had on their children’s lives and future experiences (eg, the impact of limb amputation). These outcomes featured more strongly for parents in the phase 1 FiSh and Fever studies. Only one phase 2 study participant indirectly referred to long-term effects. This suggests that priorities may evolve with the passage of time after a severe illness.

#### Reduced need/time spent on treatments and mechanical support

A reduction in the number of machines supporting their child, including duration of or need for mechanical support was described (21/85 parents, 25%) as an important short term outcome. This included time on machines (eg, ventilator and extracorporeal membrane oxygenation ECMO) and medications (eg, morphine).

#### Length of hospital stay

Seventeen parents (17/85, 20%) discussed length of stay in hospital as an important outcome to measure. Six parents (three in FiSh, three in Fever) prioritised length of intensive care stay.

### Survival

Survival was often prioritised by bereaved parents over all other outcomes yet was rarely described by parents of recovered children in FiSh and phase 1 of Fever. We amended the Fever phase 2 topic guide to explore all parents’ views on survival as an outcome after they had prioritised outcomes. Following prompting, an additional seven parents described survival as the most important outcome measure for the proposed RCT and future-related trials. Reasons why they had not initially mentioned survival included death either not being something they had wished to consider or had not considered as their child had survived (see table 3). Others had not perceived their child’s condition to be life threatening (eg, bronchiolitis).

**Table 3:** Reasons why survival had not been initially prioritised by parents

Theme	Illustrative quote
Death was not considered as an outcome	‘*I wouldn’t have necessarily connected the two, that, um, that fever could be so, um, um, so linked to, to something so, you know, grave but yeah, um, I guess anything can happen, can’t, can’t it?*’ (P73, interview, father, Fever phase 2).
Don’t want to think of death as a possibility	‘*It’s just something that you don’t, you don’t let enter your mind. So on reflection, when you’re thinking back, you don’t think of it. I can see that is, er, should be number one on the list, definitely*’ (P53, interview, father, Fever phase 2).
Because child survived so death not considered	‘*I think because, you know, she has survived it all through, that sort of goes to the back of your mind again, you know. You sort of don’t think that, you think of oh what… but now, you know, now you think about it, you just, yeah, you don’t think of that. I don’t know, it’s strange*’ (P78, interview, mother, Fever phase 2).
*Not an option – my child was going to survive*	‘*“There’s no option. I’ve lost a nephew, erm, five or six years ago, so, obviously, going into hospital and having them on the machines was, er… like brought back lots of memories, when they’re in intensive care, there’s only two ways out of it. You either don’t come out or you come out, so like I said to my wife, we’re definitely getting them out, or my son, because he was the only one ill at that time.* (P55, interview, father, Fever phase 2).
*Assured no risk of death*	‘*As soon as we got there, they said, we treat this all the time, she’ll be fine. So I didn’t really ever think at that point that she wasn’t going to survive…*’ (P79, interview, mother, Fever phase 2).

## Discussion

Our findings provide new insight into parent-prioritised outcomes for paediatric emergency medicine or intensive care trials investigating treatments for severe infection. In addition to survival, parents-prioritised short-term outcomes include: organ and physiological functioning; their child looking and/or behaving more like their normal self; and length of time on treatments or mechanical support. Longer term prioritised outcomes included physical and developmental effects of illness on the child.

Consistent with outcomes used in previous trials in this condition,^23–25^ parents prioritised improvements in their child’s organ and physiological functioning as an indicator of treatment efficacy and recovery. Specifically, parents favoured the alleviation of core infection-associated symptomatology, such as heart rate, temperature and respiratory rate, increase in blood pressure and hydration. Parents frequently referred to their observation of monitoring displays and treatments their child received. Linked to these outcomes was time to liberation from therapeutic treatments and machines, particularly mechanical ventilation. These outcomes were important, as a reduction in their number and required usage (eg, time) was viewed as an indication that their child was getting better.

The ideal outcome measure in septic shock has been described as ‘patient centred, easily measured, and clinically important’,^6^ while longer term outcomes are often not prioritised due to the practical and financial costs of collecting such data. If trial findings are to be impactful in ensuring patient centred healthcare^26 27^ and influencing policy and practice, then they must be relevant and important to patients and families.^11 28^ Long-term effects of severe infection, such as physical and developmental consequences, and the short-term outcome of ‘looking and behaving more like normal self’ are not easily measured and would rely on parent reports. Nevertheless, this should not preclude their consideration or inclusion. Indeed, ‘looking and behaving more like normal self’ was the second most commonly prioritised outcome across both studies. To our knowledge, there is no validated tool for this outcome. Scales such as the Pediatric Overall Performance Category,^15^ Children’s Critical illness Impact Scale^29^ and Functional Status Score^30^ could help measure some of the functional morbidity and behavioural outcomes described. However, they do not capture parents’ perspectives on outcomes observed during their child’s PICU stay. Further work is needed to define and develop this parent prioritised outcome measure.

Survival was prioritised by bereaved parents over all other outcomes. However, in many interviews with non-bereaved parents, survival was only prioritised after specific prompting. Our findings showed that parents had either not perceived their child’s life to be at risk, had high expectations of healthcare treatments or had not wished to consider the death of their child as a possible outcome. This suggests that some parents either did not fully comprehend the severity of their child’s illness,^31^ or they had developed adaptive coping behaviours to seek agency over the stressful situation by not considering death as a possibility.^32 33^ However, as mortality rates related to severe infection are declining in most affluent countries, morbidity-related outcomes may be the most appropriate focus for future trials investigating severe childhood infections.^6 34^

Interestingly, the point at which parents were asked about outcomes in the course of their child’s illness appeared to influence prioritisation. This suggests the need for the development of a core outcome set to include patients/family members with a range of relevant experiences for that condition and to include participants at different time points in the course of their, or their child’s, illness. This list of parent-centred outcomes can be used in future core outcomes set consensus work (see online S3 table 2) involving all key stakeholders (eg, clinicians, parents and children). Consensus methodology could also be used to identify the optimal duration of follow-up in these studies, including which outcomes to measure at short-term and longer term time points.

### Strengths, limitations and future implications

Our findings contribute to an important and under-researched area and demonstrate the value of using qualitative methods to explore parents’ perspectives on outcome measures. Synthesis of study findings was strengthened by harmonised study designs, topics guides and processes, which were developed and conducted by the same expert team. Recruitment in each phase was conducted until data saturation was reached,^16 17^ and both bereaved and non-bereaved parents with varying experience of their child being treated for severe infection were included, providing new insight into how views on outcomes may change with illness severity and trajectory. As the majority of FiSh and Fever participants were infants (eg, in Fever 64% were <1 year old), our insight into important outcomes for paediatric septic shock is limited to the views of parents. Future research is required to explore children’s views on important outcomes in emergency and critical care trials.

The list of outcomes compiled from the FiSh literature review was presented to parents in the phase 1 studies. Use of a list may have influenced their views on outcomes. Nevertheless, common themes were identified within and across phases of FiSh and Fever studies and similarities between suggested outcomes and the predefined set were evident. Finally, more interviews were conducted with phase 1 parents (n=46) compared with phase 2 parents (n=39), which may have biased the order of combined outcomes towards phase 1 parents’ priorities.

## Conclusions

Our findings provide insight into short-term and long-term outcomes prioritised by parents to inform the design of future trials investigating treatments for paediatric suspected infection as well as core outcome set development work. In addition to survival, parents prioritised organ and physiological functioning their child looking and/or behaving more like their normal self; length of time on treatments or mechanical support, and long term and effects on child development.
