# Supplementary material for: Parents’ prioritised outcomes for trials investigating treatments for paediatric severe infection: a qualitative synthesis
Source: Arch Dis Child. 2019 Jun 7;104(11):1077–82. doi: 10.1136/archdischild-2019-316807 (PMC6837249; doi:10.1136/archdischild-2019-316807)
Supplement: Supplementary data [file archdischild-2019-316807supp001.pdf]

Table 1. Example questions and prompts used to explore parents views on important outcomes in FiSh and Fever studies

Phase 1 interview example outcome related questions and prompts

As we have discussed, in the (FiSh/Fever) Trial we want to find out whether critically ill children with symptoms of severe infection should be *given treatments for fever at a higher temperature, up to 40.0°C, than usual, up to 37.5°C. FiSh: should be treated with less fluid (10 millilitres (ml) per kilogram (kg)) than is currently recommended (20 ml per kg).*

To do this we will collect information on: *(read through outcome measures list*). By collecting information on these main things we hope to find out which treatment for fever should be used in the future. These are called outcome measures. However, these outcomes have come from research papers and don't really give us much information on how children or families feel, or what is important to them. It is important that we include outcome measures that matter to children and their families.

1. Thinking about your experience of your child being admitted for a severe infection- what would you hope the fluid bolus would do to help your child? (*Prompt: what effect would the treatment have to be useful?*)
2. What would you be looking for as an indicator that your child was getting better?
3. What do you think about the outcome measures (*re-cap measures identified by systematic review*) is there another outcome measure that you think is important to families which we should be collecting information about in the FiSh/Fever Study? (*prompt: present identified outcome measures based on responses to Q1 and Q2*)

Phase 2 interview example outcome related questions and prompts

Thinking about your experience of your child being admitted for a severe infection…

1. what would you hope the fever treatment would do to help your child? (*Prompt: what effect would the treatment have to be useful?*)
2. What would you be looking for as an indicator that your child was getting better?
3. Explore whether parents were looking at symptoms vs. machines/figures. Given what we have talked about if this study goes to full trial, what outcomes do you think we should measure?
4. You mentioned X Y Z which would you say are the most important? *(Prompt if they don't list, if they do just check ranking- Could you list them in order?)*
5. Fever study only (if applicable): like a number of families we have interviewed you haven't mentioned survival? Is there a reason for that? (Prompt if they see it as important then see if they would like to look at the ranked list again)
