# Supplementary material for: Parents’ prioritised outcomes for trials investigating treatments for paediatric severe infection: a qualitative synthesis
Source: Arch Dis Child. 2019 Jun 7;104(11):1077–82. doi: 10.1136/archdischild-2019-316807 (PMC6837249; doi:10.1136/archdischild-2019-316807)
Supplement: Supplementary data [file archdischild-2019-316807supp002.pdf]

Box 2: Glossary of terms

	Description
Data saturation	The point at which no new key themes are identified in the analysis of data. Interview or focus group conduct usually stops when the research team identifies that data saturation point has been reached.
Interpretive	Interpretative research is based on the assumption that social reality is not singular or objective, but shared by individual experiences and social contexts.
Inductive	Inductive analysis generates themes from the data with no prior assumptions or framework.
Iterative process	Repeated iterations of reading data (e.g. interview manuscripts) and interpreting meaning (e.g. as part of the constant comparative approach).
Constant comparative analysis	The repeated comparison of information from data collection and emerging themes or theory.
Coding framework	Coding is a way of indexing or categorizing the analysis of qualitative data, such as manuscript text. As part of the analysis process coded text is organised into a framework of themes or ideas.
Core Outcome Set	Core Outcome Sets are an agreed standard sets of outcomes that represent the minimum outcomes that should be measured and reported in clinical trials of a specific condition. For further information please see the COMET (Core Outcome Measures in Effectiveness Trials) initiative: http://www.comet-initiative.org/

*Descriptions adapted from Robson, C (2002) Real World Research. London, Blackwell publishing and the COMET website, accessed 05/03/19
