# Supplementary material for: Parents’ prioritised outcomes for trials investigating treatments for paediatric severe infection: a qualitative synthesis
Source: Arch Dis Child. 2019 Jun 7;104(11):1077–82. doi: 10.1136/archdischild-2019-316807 (PMC6837249; doi:10.1136/archdischild-2019-316807)
Supplement: Supplementary data [file archdischild-2019-316807supp003.pdf]

Table 2 Outcomes prioritised by parents in the FISH and Fever studies

FiSh phase 1 outcomes (n = 21 interviews)			FiSh phase 2 outcomes (n = 20 interviews)			Fever phase 1 outcomes (n = 25 interviews)			Fever phase 2 outcomes (n = 19 interviews)			Combined list of prioritised outcomes (n= overall number o parents who prioritised this outcome)
Outcome description	Number of parents who prioritised outcome	Overall number (including indirect descriptions)	Outcome description	Number of parents who prioritised outcome	Overall mentions (including indirect descriptions)	Outcome description	Number of parents who prioritised outcome	Overall mentions (including indirect descriptions)	Outcome description	Number of parents who prioritised outcome	Overall mentions (including indirect descriptions)	
long-term effects (e.g. health and development)	14	16	*Improvement in organ and physiological function*	12	12	*long-term effects (e.g. health and development)*	8	11	*Improvement in organ and physiological function*	10	12	*Improvement in orga and physiological function (n= 38)*<br><br>*Looking and behavin more like normal self (n= 33)*<br><br>*long-term effects (e.g health and development) (n=22)*<br><br>*Reduce need/time spent on treatments and mechanical support (n= 21)*<br><br>*Length of stay (n=17)*<br><br>*Survival (n=16 / n=22 when prompted)*
Improvement in organ and physiological function	13	20	*Looking and behaving more like normal self*	10	12	*Looking and behaving more like normal self*	5	9	*Looking and behaving more like normal self*	7	1	
Looking and behaving more like normal self	11	11	*Reduced need/time spent on treatments and mechanical support*	3	3	*Length of time on mechanical ventilation*	7	8	*Not in pain and/or discomfort*	6	0	
Survival	7	9	*Practitioner demeanour*	1	1	*Length stay*	6	6	*Length of time on mechanical ventilation*	5	0	
Reduced need/time spent on treatments and mechanical support	6	8	*Length of stay*	0	1	*Improvement in organ and physiological function*	3	10	*Length of stay*	5	0	
Practitioner demeanour	4	4	*Survival*	1	1	*Effect on family*	2	5	*Number of readmissions to hospital*	2	0	
Length of stay	6	6	*Long term effects including health and development*	0	1	Survival	2	4	Survival	7 (14 when prompted)	0	
Time to recovery	3	4										
